# Fulminant Pancreatitis Due to Disseminated Histoplasmosis: Case Report and Literature Review

**DOI:** 10.7759/cureus.12168

**Published:** 2020-12-19

**Authors:** Abuzar A Asif, Moni Roy, Sharjeel Ahmad

**Affiliations:** 1 Internal Medicine, University of Illinois College of Medicine at Peoria, Peoria, USA

**Keywords:** necrotizing pancreatitis, disseminated histoplasmosis, histoplasma capsulatum, immunocompromised, histopathology, granuloma

## Abstract

Acute pancreatitis (AP), an inflammatory disease of the pancreas, is clinically classified into mild AP, moderately severe, and severe based on local complications and presence of organ failure. Histoplasmosis, caused by the dimorphic fungus Histoplasma capsulatum, typically presents with pulmonary disease. Extrapulmonary disease/disseminated histoplasmosis can affect the gastrointestinal tract, with only a few reported cases of pancreatitis secondary to the above. We describe a rare case of a young woman who presented with necrotizing pancreatitis secondary to histoplasmosis. The etiology of pancreatitis remained unclear throughout her hospital stay despite extensive workup performed. Diagnosis of disseminated histoplasmosis was based on autopsy findings.

## Introduction

Acute pancreatitis (AP), an inflammatory disease of the pancreas, presents with abdominal pain as the most common symptom. Gallstones and alcohol consumption are the most common causes of pancreatitis. However, in the absence of these risk factors, other less common causes need to be considered [1,2]. Infections are thought to be responsible for 10% of pancreatitis cases; mostly due to viruses, bacteria, and parasites. There exists a scarcity in the documentation of fungal causes of pancreatitis [3-5]. Histoplasma capsulatum, classically manifests as pulmonary disease, while extra-pulmonary manifestations, especially disseminated histoplasmosis, occurs in immunocompromised patients. The gold standard for the diagnosis of histoplasmosis involves histopathologic examination and fungal cultures [6]. Necrotizing pancreatitis is a late complication of pancreatitis and is atypically a manifestation of disseminated histoplasmosis. We present a rare case of necrotizing pancreatitis due to Histoplasma capsulatum, diagnosed on autopsy.

## Case presentation

A 41-year-old female with a history of recurrent AP due to hypertriglyceridemia, chronic steroid use for interstitial cystitis, presented with acute severe generalized abdominal pain. The patient had been hospitalized twice in the last three months for AP in the setting of hypertriglyceridemia (> 5000 mg/dL), treated with plasmapheresis, and subsequently was started on fenofibrate. She presented with severe generalized abdominal pain, worst in the epigastric region, radiating to the back. The patient reported the use of approximately 4 grams of naproxen daily for 4-5 days prior to presentation for treatment of leg pain. Other history was significant for right atrophic kidney, type 2 diabetes mellitus (treated with metformin), hypothyroidism, chronic kidney disease, hysterectomy, and inguinal hernia repair. The patient denied recent travel outside the United States, any history of incarceration, recent illnesses, or sick contacts. She quit smoking five years ago, did not drink alcohol or use any illicit drugs.

Physical exam on presentation revealed an afebrile, tachycardic (heart rate 118 bpm), normotensive, morbidly obese (body mass index (BMI) 41.20 kg/m²) female, with a distended, non-tympanic, diffusely tender abdomen which was dull to percussion. Murphy’s sign was absent and there was no rebound tenderness.

On admission, laboratory examination of blood reported leukocytosis, anemia, hypercalcemia, increased creatinine, elevated lipase levels, hypertriglyceridemia, and increased inflammatory markers including C- reactive protein and erythrocyte sedimentation rate. Table 1 demonstrates pertinent laboratory examination findings.

**Table 1:** Pertinent laboratory examination results

Component	Value	Reference range	Unit
White blood cell	16.96	4.00 - 12.00	10^3^/mcL
Hemoglobin	11.9	12.0 - 15.8	g/dL
Platelets	433	140 - 440	10^3^/µL
Aspartate aminotransferase	48	5 - 34	U/L
Alanine aminotransferase	46	0 - 55	U/L
Alkaline phosphatase	84	40 - 150	U/L
Total Bilirubin	1.2	0.2 - 1.2	mg/dL
Calcium	11.3	8.4 - 10.2	mg/dL
Albumin	4.1	3.5 - 5.0	g/dL
Creatinine	4.89	0.60 - 1.00	mg/dL
Lipase	700	8 - 78	U/L
Triglycerides	644	<150	mg/dL
C- reactive protein	36.96	<0.50	mg/dL
Erythrocyte sedimentation rate	116	0 - 20	mm/h

**Figure 1:**
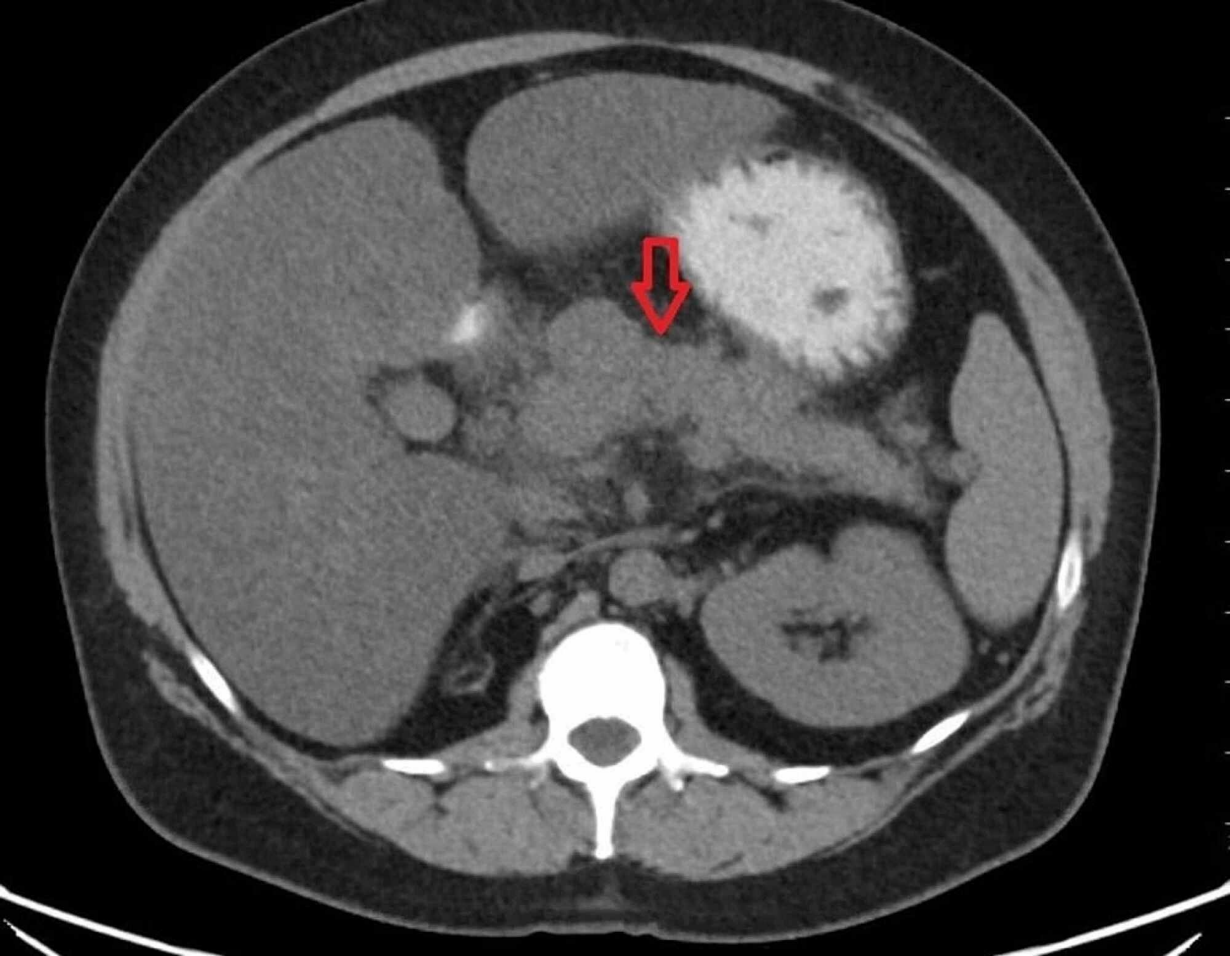
CT scan of abdomen showing pancreatitis and peripancreatic fluid collections

Chest X-ray revealed low lung volumes with atelectasis in the lung bases. Abdominal computerized tomography (CT) showed pancreatitis with multiple low-density peri-pancreatic fluid collections with the largest measuring 17 cm in greatest dimension (Figure 1). Intravenous contrast was not used to prevent worsening of acute kidney injury; therefore, capsular enhancement, pancreatic necrosis, and a pseudo aneurysm in the region of pancreas were not well evaluated. Blood cultures obtained on admission remained negative.

The patient was started on intravenous fluid resuscitation and analgesics for the treatment of AP. A bedside index for severity in acute pancreatitis (BISAP) score of 2 was calculated in order to predict her mortality risk. However, the patient’s renal function continued to worsen and she was eventually started on hemodialysis.

During the hospital stay, the patient’s abdominal pain continued to worsen, despite being on high dose analgesic medications. An episode of hematemesis prompted an esophagogastroduodenoscopy (EGD) which revealed Mallory-Weiss tear and severe erosive esophagitis; esophageal brushings sample grew Candida on culture for which she was started on fluconazole and proton pump inhibitor therapy.

**Figure 2:**
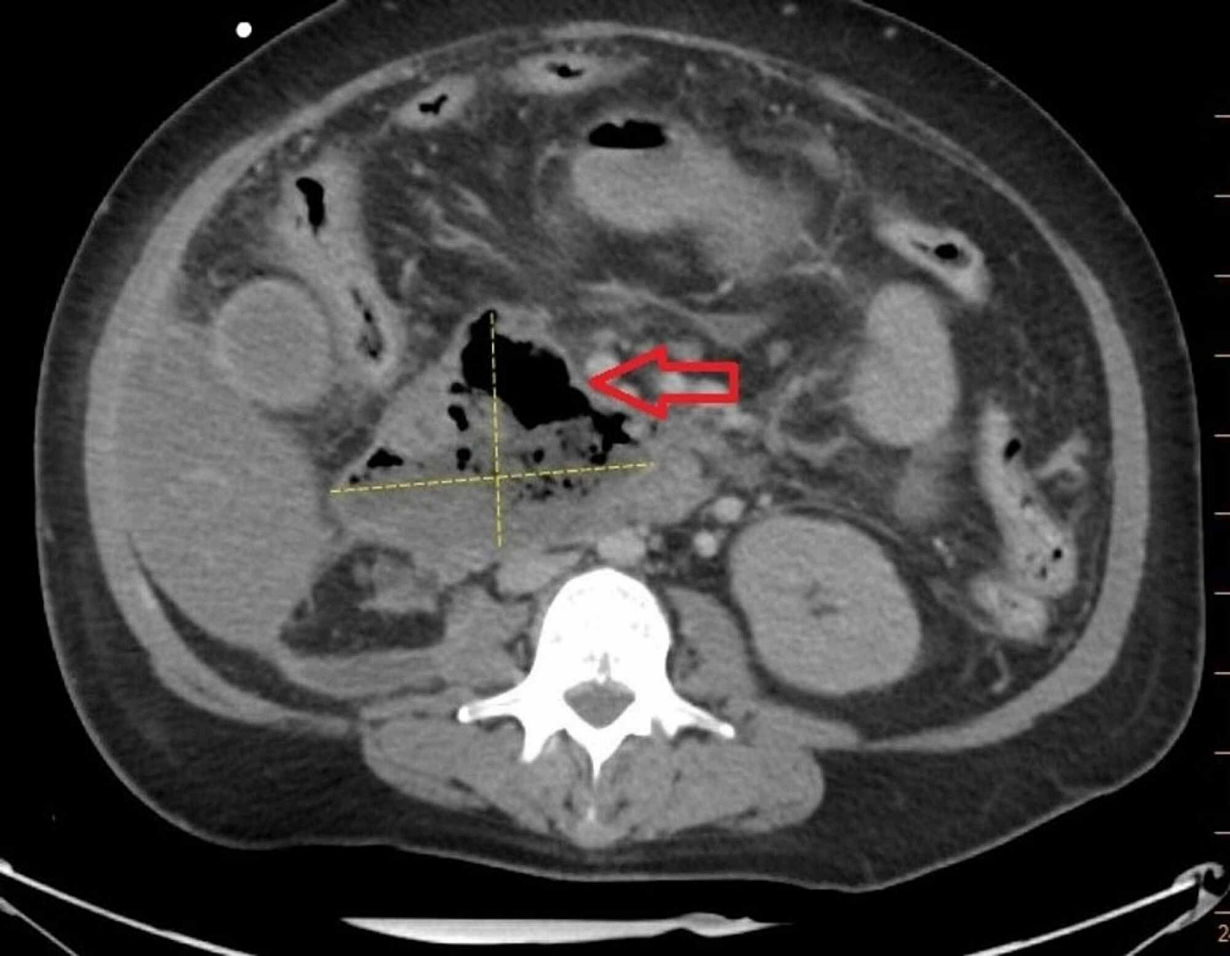
CT scan of abdomen showing pancreatic abscess

A repeat CT abdomen/pelvis was performed due to persistent pain and abdominal distention and showed new peri-hepatic and peri-colic collections (that were communicating with peri-pancreatic fluid) which were then drained percutaneously. Cultures from the drain grew Escherichia coli. Acute necrotizing pancreatitis with superimposed infection was suspected for which the patient was treated with intravenous meropenem. A follow-up CT eight days later showed complete drainage of necrotic collection inferior to the left hepatic lobe and decrease in size of necrotic collection posterior to the head of pancreas, but also showed air within the collection concerning abscess formation (Figure 2).

**Figure 3:**
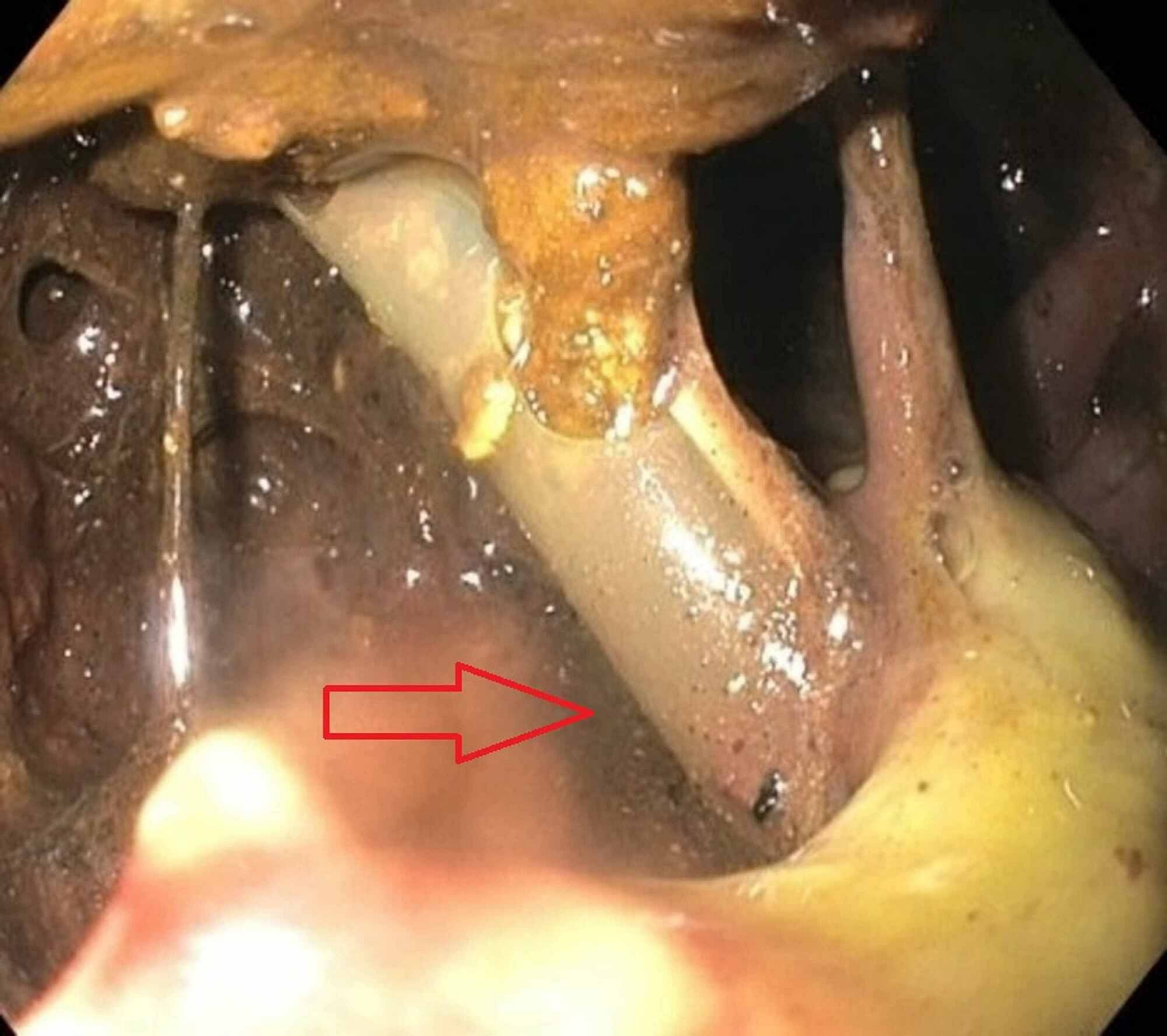
Esophagogastroduodenoscopy (EGD) showing pancreaticoduodenal fistula, with percutaneous drain visualized through the fistula

The patient’s drain output was noted to be bilious in appearance prompting a hepatobiliary iminodiacetic acid (HIDA) scan which suggested a bile duct leak and raised concern for fistula formation. A repeat EGD confirmed a 5-6 cm fistula extending from the duodenum (D2) into the necrotic cavity. The tip of the percutaneous drain was also visualized through the fistula (Figure 3).

The patient underwent extensive debridement and necrosectomy of the necrotic collection on two different occasions. Due to the patient’s worsening nutrition status, a jejunostomy tube was placed and she was started on tube feeds. Despite all aggressive measures, the patient’s functional status continued to decline and she was transitioned to do-not-resuscitate status. Unfortunately, she had acute decline in her status with hypotension, and passed away from suspected septic shock. 

**Figure 4:**
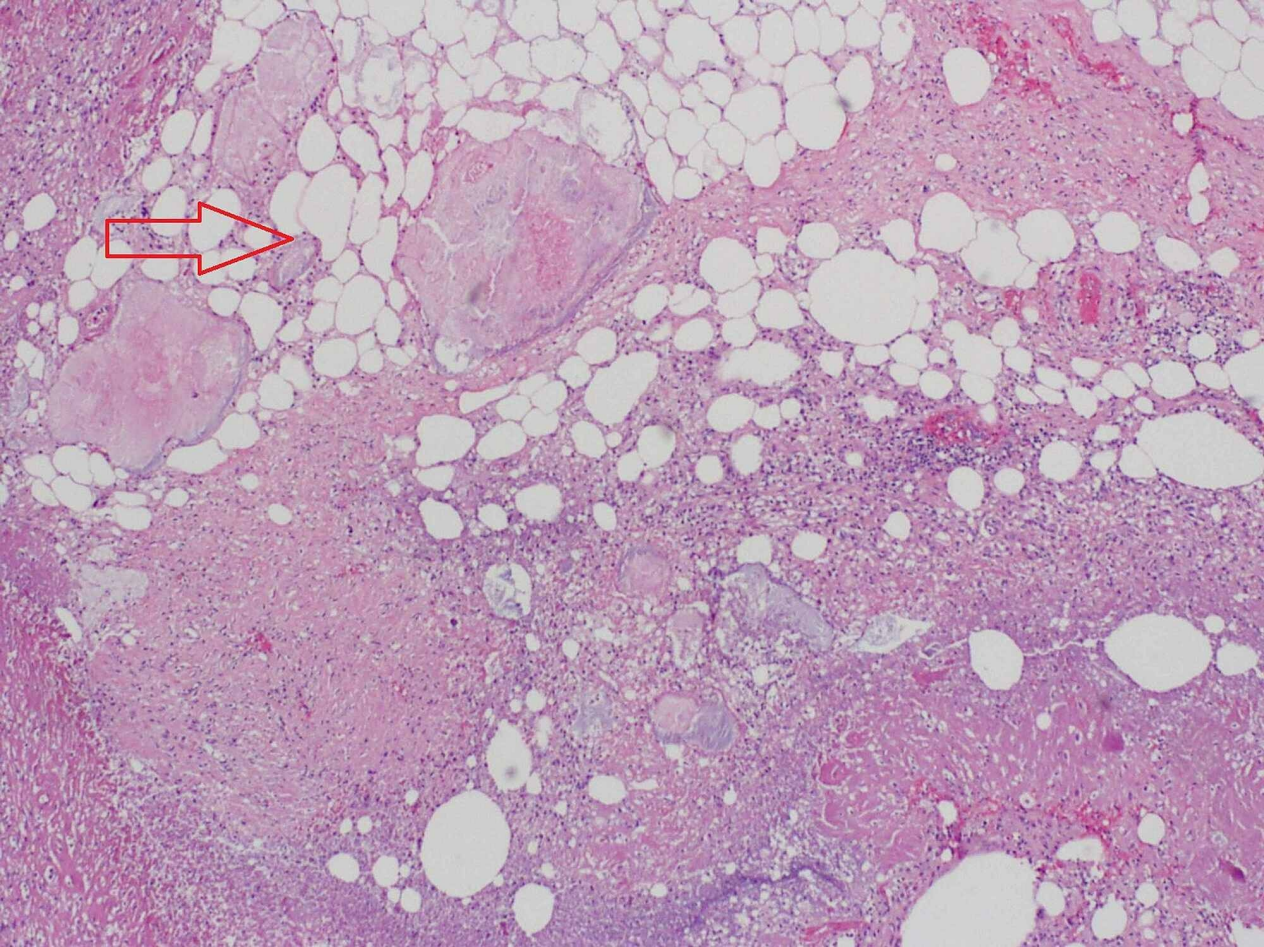
Hematoxylin and eosin (H&E) stain – fatty necrosis in pancreatic tissue (100x)

**Figure 5:**
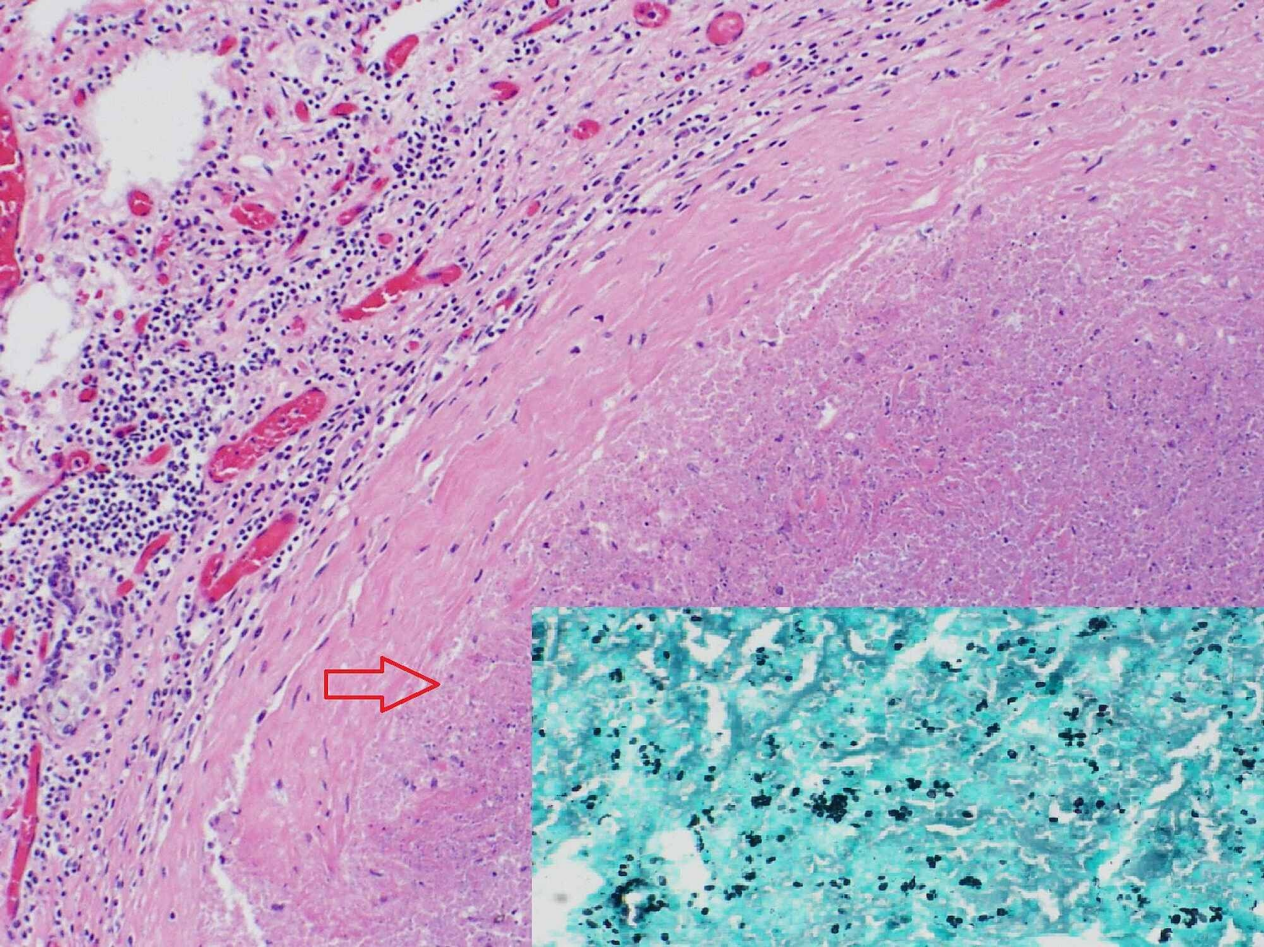
Hematoxylin and eosin (H&E) stain – necrotizing granuloma of the lung (100x); inset- GMS staining of lung granuloma showing budding yeast (400x) GMS: Grocott-Gomori's methenamine silver

A post-mortem autopsy report revealed acute tubular necrosis of kidneys, severe necrotizing pancreatitis (as seen in Figure 4), retroperitoneal abscess, acute necrotizing cholecystitis, and necrotizing granulomatous inflammation with Histoplasma capsulatum (identified by Grocott-Gomori's methenamine silver (GMS) staining) involving right and left lungs, spleen, liver and mediastinal lymph nodes (Figure 5). These findings were consistent with the diagnosis of disseminated histoplasmosis.

## Discussion

AP typically presents with abdominal pain, most prominent in the epigastrium. Diagnostic criteria for pancreatitis includes any two of the following three factors: acute severe epigastric pain, usually radiating to the back; elevated pancreatic enzymes (lipase or amylase) to three times the upper limit of normal; and characteristic findings on imaging [1].

In addition to gallstones and alcohol consumption, other less common causes of pancreatitis warrant consideration. Hypertriglyceridemia has been associated with AP, with a reported incidence of 2%-4%, typically when triglyceride levels exceed 1000 mg/dL [2]. Infectious agents, medications, and metabolic causes such as hyperparathyroidism and hypercalcemia, are other rare causes of pancreatitis [3]. Our patient denied any alcohol use and absence of cholestatic pattern on liver function tests along with the normal appearance of common bile duct on CT, ruled out gallstones as a cause of pancreatitis. Although hypertriglyceridemia was possibly the cause of prior episodes of pancreatitis in our patient, a triglyceride level of < 1000 was unlikely the cause of the current presentation.

Infections are thought to be responsible for 10% of pancreatitis cases; mostly due to viruses (hepatitis B virus, mumps virus, cytomegalovirus, herpes simplex virus, human immunodeficiency virus), bacteria (leptospirosis, Mycoplasma pneumoniae), and parasites (ascarislumbricoides). Pancreatitis due to fungal organisms is rarely reported in the literature. Candidal infection complicating necrotizing pancreatitis rather than being etiology of pancreatitis, has been well documented in medical literature [4]. Aspergillus infection leading to inflammation, infarction, and necrosis of pancreas has also been described [5].

Pancreatitis due to Histoplasma capsulatum is extremely rare. Immunocompromized individuals are at high risk of acquiring disseminated histoplasmosis. The most common sites of dissemination include the central nervous system, adrenals, gastrointestinal tract, and reticuloendothelial system (bone marrow, liver, spleen, and lymph nodes) [6]. Review of literature using PubMed and Google scholar revealed five other published case reports on histoplasmosis involving the pancreas (Table 2) [7-11]. 

**Table 2:** Cases reports of pancreatic Histoplasma involvement DH: disseminated histoplasmosis; CBD: common bile duct; EUS: endoscopic ultrasound; FNA: fine-needle aspiration; PAS: periodic acid-Schiff.

Year	Author	Age / Gender	Geographic location	Immunity status	Clinical features	Diagnosis / comments	Disseminated or isolated histoplasmosis	Treatment / antifungal therapy	Reference
2017	Harris CE et al.	69 / F	Wisconsin	Immunosuppressed	Fever, fatigue, abdominal pain, diarrhea, epigastric tenderness	Elevated lipase and CT abdomen/ pelvis suggestive of pancreatitis, histoplasma urine antigen weakly positive, DH confirmed with positive blood cultures and bone marrow biopsy	DH	Liposomal amphotericin B for 7 days later transitioned to oral itraconazole for a total of 9 months.	[7]
2015	Aggarwal A et al.	37 / F	Minnesota	Immunocompetent	Painless jaundice	Elevated transaminases, hyperbilirubinemia. MRI abdomen reported pancreatic head mass with amorphous calcification externally compressing CBD and portal vein. Pancreatic histoplasmosis confirmed on serology and histopathology of resected specimen.	Isolated pancreatic histoplasmosis	Voriconazole for 3 months. (unable to tolerate itraconazole)	[8]
2014	Choudhary NS et al.	61/F	India	Immunocompetent	Vomiting, weight loss.	CT abdomen showed pancreatic head mass causing duodenal obstruction. EUS guided FNA with periodic acid-Schiff staining showed round yeast forms with histoplasma morphology, indicating pancreatic histoplasmosis.	Isolated pancreatic histoplasmosis	Itraconazole (unspecified duration)	[9]
2013	Subbalaxmi et al.	56 / M	India	Immunocompetent	Hematuria, epigastric pain and tenderness	Elevated amylase and clinical findings suggestive of pancreatitis. Bone marrow biopsy performed for persistent thrombocytopenia reported histoplasmosis on BM aspirate histology and culture.	DH	Amphotericin B deoxycholate for 2 weeks followed by oral itraconazole for 12 months Epigastric pain resolved after course of amphotericin B.	[10]
1994	Johnston AW et al.	70/ M	-	Immunocompetent	Malaise, weight loss, low grade fever, hypotension, HSM. Initially diagnosed with chronic DH involving the adrenal glands treated with amphotericin B.	10 year follow up showed pancreatic and adrenal abscess on autopsy. On samples from the abscesses Grocot and PAS stain tested positive for non-viable histoplasma.	DH, case of possible relapse of chronic DH.	-	[11]

Pancreatitis due to disseminated histoplasmosis has also been reported in some large center studies. In a retrospective analysis, Wheat et al. identified risk factors associated with severe histoplasmosis in acquired immunodeficiency syndrome (AIDS) patients during the 1988-1995 outbreak in Indianapolis. A total of 145 of 155 total cases were identified as disseminated histoplasmosis, out of which only one case manifested as pancreatitis [12]. A single-center study by Putot et al. aimed to describe the clinical presentation, diagnostic modalities, and factors associated with one-year mortality in human immunodeficiency virus (HIV) positive individuals with disseminated histoplasmosis, between the years 2002 to 2012, at a large tertiary care facility. In this study, only three out of 82 cases had pancreatitis associated with disseminated histoplasmosis [13]. In a multicenter study to characterize the histopathologic variety seen in gastrointestinal and hepatic histoplasmosis, Lamps LW et al. reported 52 cases of gastrointestinal histoplasmosis confirmed on surgical pathology and autopsy, of which, three cases of pancreatitis were recognized [14].

In our case, AP at presentation was complicated by pancreatic and peri-pancreatic necrosis followed by the formation of pancreato-duodenal fistula. Multiorgan failure eventually led to our patient’s demise. However, the exact etiology remained unclear until an autopsy identified necrotizing pancreatitis in the setting of disseminated histoplasmosis. Chronic steroid use by our patient for interstitial cystitis likely contributed to immunosuppression and increased her risk of acquiring histoplasmosis.

Disseminated histoplasmosis presents with fever, malaise, anorexia, and weight loss. When severe, it can present as sepsis syndrome with hypotension, disseminated intravascular coagulation (DIC), respiratory and renal failure [9]. Although gastrointestinal involvement is common in disseminated histoplasmosis, it usually presents with vague abdominal symptoms such as diarrhea and abdominal pain or remains asymptomatic. It therefore can delay diagnosis and treatment of histoplasmosis, when more common differentials of these symptoms are considered and investigated [15,16].

Laboratory findings including pancytopenia, elevated alkaline phosphatase, elevated erythrocyte sedimentation rate, C- reactive protein, high ferritin levels, and high lactate dehydrogenase levels, are non-specific but highly suggestive for disseminated histoplasmosis in the setting of appropriate clinical presentation [15].

The gold standard for diagnosis of histoplasmosis involves histopathologic examination and detection on fungal cultures. The presence of caseating or non-caseating granuloma is characteristic. Histoplasma capsulatum appears as narrow-based budding yeast on GMS stain and periodic acid-Schiff (PAS) stain and is predominantly intracellular within macrophages. Fungal cultures require four to six weeks to grow and demonstrate Histoplasma [6,17]. Post-mortem autopsy findings in our patient revealed defined necrotizing and confluent granulomas involving several organs. Biopsy of the pancreas showed severe necrosis; therefore intracellular Histoplasma could not be appreciated. Based on autopsy findings of diffuse histoplasmosis involving multiple other organs and review of literature showing histoplasmosis causing pancreatitis, we think our case of fulminant pancreatitis most likely was due to disseminated histoplasmosis.

## Conclusions

Histoplasma capsulatum, a rare etiology of AP, can lead to life-threatening complications if missed at presentation. Immunocompromised individuals are most commonly at risk but cases in immunocompetent patients have also been reported, and therefore should not be overlooked. Increasing utilization of polymerase chain reaction (PCR) assays and next-generation microbial cell-free DNA sequencing may help detect Histoplasma capsulatum earlier than traditional time-consuming techniques. We suggest keeping a high index of suspicion for disseminated histoplasmosis in patients with unexplained pancreatitis presenting from endemic regions. In retrospect, early testing for disseminated histoplasmosis, in our patient who was from the Midwest region of United States (endemic for histoplasmosis), could have prevented a poor outcome.
